# Concealment of trauma and occupational accidents among Fukushima nuclear disaster decontamination workers: A case report

**DOI:** 10.1002/1348-9585.12123

**Published:** 2020-05-05

**Authors:** Toyoaki Sawano, Hayato Tanaka, Daiki Watanabe, Akihiko Ozaki, Manabu Tsukada, Yoshitaka Nishikawa, Hiroaki Saito, Yuki Shimada, Tomohiro Morita, Hiromichi Ohira, Masaharu Tsubokura

**Affiliations:** ^1^ Department of Surgery Minamisoma Municipal General Hospital Fukushima Japan; ^2^ Department of Public Health Fukushima Medical University School of Medicine Fukushima Japan; ^3^ Department of Surgery Sendai City Medical Center Miyagi Japan; ^4^ Department of Breast Surgery Jyoban Hospital of Tokiwa Foundation Fukushima Japan; ^5^ Research Center for Community Health Minamisoma Municipal General Hospital Fukushima Japan; ^6^ Department of Internal Medicine Soma Central Hospital Fukushima Japan; ^7^ Department of Health Informatics School of Public Health Kyoto University Kyoto Japan; ^8^ Department of Gastroenterology Sendai Kousei Hospital Miyagi Japan; ^9^ Department of Neurosurgery Minamisoma Municipal General Hospital Fukushima Japan

**Keywords:** decontamination worker, environmental health, Fukushima, nuclear disaster, socioeconomic status, workspace safety

## Abstract

**Objectives:**

Limited information exists concerning occupational risks in decontamination work after the Fukushima Daiichi Nuclear Power Plant (FDNPP) accident. Workers involved tend to be migrant workers, face various health risks, and are usually from a low socioeconomic background and generally have difficulty in finding employment. We report a specific case to illustrate the way these workers tend to get injured during working hours and draw attention to the problems arising.

**Case presentation:**

A 59‐year‐old Japanese male decontamination worker was referred to our emergency department after a fall while he was working in an Exclusion Zone surrounding the FDNPP. He was blind in his right eye. He was diagnosed with traumatic multiple rib fractures and a tube thoracostomy was performed. He was discharged from hospital after 7 days. Payment has been changed from “occupational accident,” which is required to be reported to the Local Labor Standards Office, to “general medical treatment” which is no obligation.

**Conclusion:**

Trauma or physical injury of any kind is an occupational hazard for workers, especially those operating in the chaotic and unpredictable environments following any disasters. Companies employing such workers and owners of any facilities or locations in which they may be working are responsible for the safety of their workers. They should provide appropriate training and should comply with all prevailing Employment Laws and follow mandatory safety regulations. If companies and authorities are in breach of any laws, ignore their responsibilities, or jeopardize the health of their workers, they should be held accountable.

## 1 INTRODUCTION

Workers engaged in potentially life‐threatening work connected with cleaning up after radioactive contamination caused by a nuclear accident needs special protection from the wide variety of dangers they may face. This is especially so as they are often migrant, poorly educated, low‐skilled personnel, usually of low socioeconomic status (SES), who, for whatever reason, have found difficulty in finding suitable employment elsewhere. After the Great East Japan Earthquake and tsunami in March 2011, the Fukushima Daiichi Nuclear Power Plant (FDNPP) suffered a major accident causing the release of dangerous radioactivity into the environment. Extensive and intensive decontamination work was launched in the radiation‐contaminated areas in Fukushima Prefecture to help control and mitigate the levels of radioactivity.^1^, ^2^ Many workers engaged in this work were hired from all over Japan due to the severe shortage of workers in Fukushima, the number of employees reaching a peak of approximately 40,000 in 2015.^3^ Recently, the number of workers in Fukushima has been decreasing annually, as has the area in need of decontamination.

It is known that decontamination workers in Fukushima usually have a low SES.^4^ Plus workers who have mild physical or psychological disability, and those who have difficulty in finding other work, have been more likely to be hired to help in the decontamination work. It is obvious these workers would be facing various health risks, including exposure to nuclear radiation, heat stroke, infectious diseases, mental health issues, insect stings, rodent bites and contaminated droppings, plus environmental hazards.^1^, ^4^, ^5^, ^6^, ^7^

We report a specific case to illustrate the way these workers tend to get injured during working hours and draw attention to the problems arising. Unfortunately, information concerning occupational risks in nuclear decontamination work is particularly scarce. For this reason, we consider it is important to publish detailed information about the decontamination worker who presented at the Minamisoma Municipal General Hospital.

## 2 CASE PRESENTATION

A 59‐year‐old Japanese male employed as a decontamination worker following the FDNPP accident was referred to our emergency department. He had a past history of right retinal detachment and, in consequence, had lost sight in his right eye. He was employed by a subcontractor of a general contractor, which operated a joint venture in decontamination work within an area designated as an Exclusion Zone due to the release of radioactivity from the stricken FDNPP. His duties included cutting bush and plants, scraping up surface soil, and packing everything into container bags for proper disposal. He worked an 8‐hour day for 5‐6 days a week. He had received occupational safety training and radiation‐specific special education, which is generally required for workers engaged in such decontamination work. He also received a semiannual ionization radiation check‐up and an annual general medical examination, as required by law.

He was working in the Exclusion Zone surrounding the FDNPP and was outfitted with appropriate clothing, including a long‐sleeve shirt, pants, and safety helmet. On the day before he was injured, he had worked as usual and had not felt ill. On the day of his accident, the weather was cloudy and visibility was not good. While cutting plants he fell from a height of 1 m due to stumbling in a gutter obscured by the vegetation. He suffered a severe blow to his chest. Although he experienced shortness of breath, he took an analgesic medication to relieve his pain and went home to recover for the rest of the day. By the next day, his chest and back pain had worsened considerably and he attended his local clinic where, following an X‐ray, he was referred to our hospital with a provisional diagnosis of rib fractures and pneumothorax.

Our emergency department found his shortness of breath had improved, his blood pressure was 148/84 mm Hg, pulse was 83 beats per minute with oxygen saturation of 95% in room air. Physical examination showed cutaneous abrasions on the right back, subcutaneous emphysema and decreased breath sound on the right side of his chest. Laboratory tests revealed only leukocytosis. Chest X‐ray and computed tomography revealed eighth‐to‐tenth right rib fractures plus right hemopneumothorax. He was subsequently admitted with a final diagnosis of traumatic multiple rib fractures and right hemopneumothorax, in order to drain the contents of the right thoracic cavity. A tube thoracostomy was placed in his right thoracic cavity and his chest was fixed by a bust band on admission day. After 5 days, the chest tube was removed, as his symptoms improved and expansion of the right lung was confirmed by chest X‐ray. He was discharged from the hospital on the seventh day after admission. He reported no recurrence of symptoms.

At first, the medical cost for this patient was conventionally registered as an “occupational” accident. However, he later requested for the case to be treated as a “general” accident. If identified as an occupational accident, he would have faced the possibility of being fired from work after his treatment. As a result, this case was handled as a general accident and he was able to continue his employment in the decontamination work.

## 3 DISCUSSION

In the present case, a male worker got injured during working hours, presenting right rib fractures, and hemopneumothorax. Trauma due to a fall is an occupational hazard in his particular line of work. In 2014, the Japan Advanced Information Center for Safety and Health reported that trauma is the third most common cause of medical attention needed by decontamination workers, accounting for about 5%, following heatstroke and insect bites. A considerable proportion of workers involved in decontamination work after the FDNPP accident may be more susceptible to accidents and work‐related injuries due to the hazardous and dangerous environments in which they are forced to operate, and because they may be from a limited education background and be of limited skill and lacking awareness of the risks they face on a daily basis.

Consequently, it is of paramount importance for decontamination work employers to comply fully with all relevant rules and regulations, especially the Labor Standard Act and particularly with regard to providing all required general safety and hazard‐related training. According to a report from the Fukushima Labor Standards Office, 47.0% of the 1,020 decontamination companies working in connection with the FDNPP incident had violated the Industrial Safety and Health Act, and, in 2016, 71.2% of them contravened the act related to the conditions of employment.^8^ One of the clearest examples was an employer that did not let their workers wear safety belts, mandatory when working in high places. Regarding the case at hand, the patient had lost sight in his right eye and, naturally, had a greater risk of injury than other workers. Questions should therefore be asked of the employer as to his suitability to be employed for such dangerous work. Quite clearly, given the circumstances, it should have been an obligation to his employer to pay significantly greater attention to the occupational hazards, particularly so in the case of employees who are in any way handicapped.

Moreover, the patient in this case sustained an injury after a fall in an Exclusion Zone surrounding the FDNPP. This area had been abandoned except for temporarily returned residents and reconstruction workers.^9^ Companies working in the area should impress on their workers that there are many hidden dangers (due to overgrowth of vegetation and human neglect) to help avoid accidents. Workers themselves must also be constantly alert while working in such chaotic and disorderly environments.

As this case was not dealt with as an occupational accident, it is important to further investigate the situation, even if the patient requested otherwise. In Japan, the costs of medical care due to occupational accidents are essentially covered by insurance purchased by employers, as stipulated in the Labor Standards Act. All such accidents, including this case, must be reported to the local Labor Standards Office. However, in reality, employers, conventionally but unofficially and illegally, often terminate the employment of workers involved in occupational accidents. Consequently, it may be common for decontamination workers, who will usually find alternative employment hard to come by, to avoid reporting or acknowledging any occupational injury. Furthermore, social determinants of health, such as low SES, among decontamination workers will also contribute to these concealments.^10^, ^11^, ^12^

Clearly existing Labor laws need to be enforced and legal action should be taken to mitigate the occupational hazards among decontamination and other workers and to protect their employment rights. Concealment of an occupational accident should not be allowed to happen. It is important that the authorities, including the Ministry of Health, Labour and Welfare, and the Tokyo Electric Power Company Holdings, intervene to enforce existing legislation and protect decontamination workers. It would also be useful to adequately register occupational accidents (past and present cases) to enable better management of this line of work and its regulations. It is also necessary to adequately educate all hospital staff, including doctors, with respect to how to recognize and deal with occupational accidents among decontamination‐related workers. This includes their legal obligations with respect to designating any injury as “occupational” or “general” or whether this can be decided by the patient. This will become of increasing importance as greater and greater burden is placed on government funds for the provision of health care, with health insurance companies paying for the former designation and the state paying for the latter.

## 4 CONCLUSION

Trauma or physical injury of any kind is an occupational hazard for workers, especially those operating in the chaotic and unpredictable environments following any disasters. Workers involved in the clean‐up after the FDNPP accident face a wide variety of occupational hazards. Companies should be made to recognize this and be responsible for preparing adequate and specific, easily understood education and proper and appropriate training for employees, to help decrease the risk of occupational accidents and injury. All relevant existing laws need to be enforced and policed to safeguard the employment rights and safety of all employees working in dangerous environments. If companies and authorities are in breach of any laws, ignore their responsibilities, or jeopardize the health of their workers, they should be held accountable.

## DISCLOSURE

*Approval of the research protocol*: The Minamisoma Municipal General Hospital Institutional Ethics Committee waived the necessity for review of Case Reports. *Informed consent*: A written informed consent was obtained from the patient for publication of this case report. *Registry and the registration no. of the study/trail*: N/A *Animal studies*: N/A.

## CONFLICT OF INTEREST

We declare that there is no conflict of interest.

## AUTHOR CONTRIBUTIONS

Sawano T., Tanaka H., and Tsubokura M. contributed to the conception and design of the research. Sawano T. and Tanaka H. drafted the article. All authors performed critical revision of the article for intellectual content, were involved in interpretation of the case and approved submission of the article.
